# Legal and policy requirements of basic health insurance package to achieve universal health coverage in a developing country

**DOI:** 10.1186/s13104-019-4618-0

**Published:** 2019-09-13

**Authors:** Ramin Hayati, Mohammad Javad Kabir, Zahra Kavosi, Peivand Bastani, Ghasem Sobhani, Hamideh Javadinasab

**Affiliations:** 1 Student Research Committee, School of Management and Medical Informatics, Shiraz University of Medical Sciences, Shiraz, Iran; 2 Health Management and Social Development Research Center, Golestan University of Medical Sciences, Gorgān, Iran; 3 Health Human Resources Research Center, School of Management and Medical Informatics, Shiraz University of Medical Sciences, Shiraz, Iran; 4 Social Determinants in Health Promotion Research Center, Hormozgan Health Institute, Hormozgan University of Medical Sciences, Bandar Abbas, Iran; 5 Secretariat for Supreme Council of Health and Food Security, Ministry of Health and Medical Education, Tehran, Iran

**Keywords:** Universal health coverage, Legal requirements, Basic health insurance package, Document analysis, Iran

## Abstract

**Objectives:**

This study has analyzed the policy-making requirements related to basic health insurance package at the national level with a systematic view.

**Results:**

All the documents presented since the enactment of universal health insurance in Iran from 1994 to 2017 were included applying Scott method for assuring meaningfulness, authenticity, credibility and representativeness. Then, content analysis was conducted applying MAXQDA10. The legal and policy requirements related to basic health insurance package were summarized into three main themes and 11 subthemes. The main themes include three kinds of requirements at three level of third party insurer, health care provider and citizen/population that contains 5 (financing insurance package, organizational structure, tariffing and purchasing the benefit packages and integration of policies and precedents), 4 (determining the necessities, provision of services, rules relating to implementation and covered services) and 2 (expanded coverage of population and insurance premiums) sub themes respectively. According to the results, Iranian policy makers should notice three axes of third party insurers, health providers and population of the country to prepare an appropriate basic benefit package based on local needs for all the people that can access with no financial barriers in order to be sure of achieving UHC.

## Introduction

Access to basic health services with no financial limit is one of the most important goals of governments [1]. Emphasis has been put on utilization of health services at the highest possible level in WHO’s report and the most countries’ constitutions [2]. WHO has recommended the use of social health insurance as an effective strategy to reduce financial barriers to access health [3]. Nowadays, health insurance is considered as a path to achieve universal health coverage (UHC). In addition to protecting financial risks, UHC covers two other dimensions: population coverage and services package [4].

Iran as a developing country in Middle East with about 80 million population and 6.8% allocation of gross domestic product (GDP) to health sector tries to move toward UHC [5]. Looking over the country’s policies, constitution of Islamic Republic of Iran articles 2 and 29 explicitly emphasized on individuals’ access to healthcare and insurance [6]. Fifth 5-year development plan of socioeconomic also considered expansion of health oriented insurance [7].

Despite the emphasis of the policies, national evidences show that lack of sustainable provision of financial resources, inequitable and inefficient services delivered by Iranian health system and lack of governance are the main challenges in achieving UHC [8], other Iranian evidences indicate that although national efforts have led to decrease the amount of out of pocket payment (OOP) from 80.5 to 59.5% from 1995 to 2014, there is a long way to achieve UHC [9]. Lack of scientific and cost effective health basic benefit package in the country may be considered as another reason for losing to achieve UHC in spite of the coverage of about 90–95% of the whole population by any of the insurance companies [10, 11].

Other evidences emphasize that non-appropriate approach and process of these national insurance companies especially in the scope of designing suitable health basic benefit package along with inappropriate allocation of financial resources to purchase health priorities can intensify the problem [12]. Iranian Health service packages are economically provided by two separate organizations; the first one is the primary healthcare package delivered by Ministry of Health, accounts for about 35–30% of the health sector’s costs. The second is the basic health insurance package, financed by insurance organizations under the supervision of the Ministry of Social Welfare [13] in which the inclusion of different services to the package has occurred through bargaining, political negotiations and health care providers’ views [10, 11].

As it is obvious, policymakers need to have access to a variety of documents as well as research papers for proposing basic benefit package [14] however, traditional methods based on political lobbying and health providers’ conflict of interest are generally used in the common process of Iranian policy-making [15, 16]. According to what was said, the present study aims to analyze legislative documents, legal and policy requirements associated with the basic health insurance package in the last 20 years to highlight the way of presenting an appropriate package toward UHC.

## Main text

### Methods

#### Study design

This is a qualitative content analysis with inductive approach conducted in 2018. Scott 4-step method included authenticity, credibility, meaningfulness and representation of the data was used to extract the relevant documents [17, 18].

#### Study population

The research population included all documents available at the legislation level since approval of the universal insurance law (1994). The period of selecting documents were, from 1 January 1994 to 31 December 2017. These documents were examined through the websites of the main organizations that have a political role in health decision making the same as: Ministry of Health [http://www.behdasht.gov.ir], Health Insurance Organization [http://www.ihio.gov.ir], Social Security Organization [http://www.tamin.ir], Management and Planning Organization [http://www.mporg.ir], Central Insurance Council [http://www.centinsur.ir], High Council of Health Insurance [http://www.mcls.gov.ir], Parliament Research Center [http://www.rc.majlis.ir], and Medical Council [http://www.irimc.org] at macro level. Documents on the sites of universities of medical sciences and health insurance and social security departments in provinces (micro level) were excluded. Table 1 shows those included documents regarding Scott method.

**Table 1:** Categories of the final studied documents

Number	Categories of final studied documents	No of documents
1	Socio-economic and cultural development plan (second, third, fourth, and fifth)	4
2	Budget law (1994–2017)	21
3	Documents of High Council of health insurance	162
4	Related rules	7
	Universal Insurance Act Organizing health act Targeting subsidies law Civil services management law Comprehensive welfare la Comprehensive veterans services law	
5	Related policies	2
	General policies of health Policies of resistance economy	
6	Other related documents	4
	Document of 20-year visions Circular of health sector evolution plan Map of health system evolution Health scientific road map	

#### Data analysis

Documents were analyzed applying both explicit and implicit methods [19–21]. In explicit analysis, emphasis was put on content analysis of the documents directly referring to the basic health insurance package. The explicit analysis was done several times by two members of the research team. After several times of reviewing, specified words were determined in Microsoft Office to be entered into MAXQDA_10_ at the next step to identify the themes and subthemes. This process continued until development of the intended content framework.

Implicit analysis included documentations involving similar concepts and issues, such as universal health coverage, health insurance, purchasing health services, and healthcare financing.

In implicit analysis approach, after reading the texts for several time, meaningful units were identified, coded, and modified by the research team to find out the final codes [22–25]. At the next step, the texts were entered into MAXQDA_10_ to identify the themes and sub-themes. This process continued until development of the intended content framework.

All stages of analysis were conducted by two members of the research team who had no conflict of interests with the subject and the selected organizations.

### Results

According to the results, three main themes were achieved during content analysis based on a theoretical framework of healthcare triangle. This triangle shows the interactions among “citizens/population” and “third-party insurer” and “healthcare providers” as the main requirements to achieve health insurance and UHC.

As Table 2 indicates, 11 related subthemes were categorized for each of the three main themes. These subthemes were synthesized through content analysis and integration of 55 final codes that were achieved explicitly or implicitly from the documents retrieved and included in the study. Followings are more clarification about each main theme and its related subthemes:

**Table 2:** The main themes and sub-themes of legal and policy requirements of basic health insurance package from 1994 to 2017

Themes	Sub themes	Final codes
Third party insurer requirements	Financing insurance package	Increasing the health share from GDP
		Providing sustainable financial resources
		Management of resources
		Financing the healthcare insurance
		Adaptation of measurements for reimbursement
		Allocation of 10% of car insurance premiums for accident injuries
	Organizational structure	Integrating all medical funds to the Medical Services Insurance
		Establishing a High Council of Health Insurance
		Defining the country’s medical treatment system
		Making comprehensive healthcare system based on referral system
	Tariffing the service package	Using actual prices and approved per capita rate for health care
		Setting yearly tariff of services by High Council of Health Insurance
		Developing healthcare services and tariffs based on evidences
	Integration of policies and precedents	Organizing integrated health insurance services based on IT and EHR
		Establishment of Iranian insurance database and elimination of overlap
		Providing principles for contracting, behaving and monitoring insurers
		The same instructions to examine medical claims
		Entering new services to approve practical guidelines by MOH
	Purchasing the benefit packages	Strategic purchasing of health services from all sectors by HIO
		Purchasing comprehensive basic and supplementary health services
		Requiring evaluation of health technologies for health interventions
		Performance of quality-based payment
		Development of clinical guidelines
		Definition framework contracts for basic health services
		Contracting providers complying with Article 17 universal insurance law
Health care provider requirements	Determining the necessities	Determination of comprehensive healthcare services package by MOH
		Defining uniform basic health insurance services for the population
		Determining of the covered basic health insurance package in three levels
		Updating uniform approach of the list of basic insurance commitments
	Provision of services	Reinforcement of competitive market to provide medical insurance
		Defining providing mechanism to the insured health sector evolution plan
		Providing insurance services based on rules/regulations of High Council
		Avoiding contract with providers aren’t interested in UHC cooperation
		Supplying the entire requirements covered by the basic insurance package
	Rules relating to implementation	Creating the necessary measures for establishment of health insurance
		Notifying mechanisms for universal and compulsory health insurance
		Cooperating between all basic insurance organizations Ministry of Health
	Covered services	Full coverage of basic health needs for members of the Community
		Reviewing the basic insurance package with health orient approach
		Paying attention to health promotion and prevention activities
		Allocating appropriate facilities for women
		Insurance coverage for infertility treatment
Citizen/population requirements	Expanded coverage of population	Basic health insurance is universal and mandatory
		Implementing the plan of mandatory insurance of wage earners
		Providing free basic health insurance to all Iranian uninsured
		Coverage of all foreigners residing in the country
		Coverage of headed households
		Coverage of residents of villages/cities with less than 20,000 people
		Free urban inpatient scheme to underserved poor populations
		Targeted advocacy to empower supported individuals and institutions
		Establishment of basic social security and health insurance
	Insurance premium	Premium per capita healthcare coverage for insured people
		Paying the share of employees premiums by executive organizations
		Using world experience as insurance calculations framework
		Defining basic medical insurance of villagers and nomads

#### Third party insurer requirements

In this axis, five sub themes are appeared as follows: Financing insurance package by main insurance organizations in the country allocating an appropriate ratio of GDP and governmental budget to health sector along with seeking sustainable financial resources as insurance premiums and taxes.

Making an obligation to improve the organizational structure of the insurance companies and determining the hierarchy of regulation setting along with correcting the mechanism of referral system for all insured people.

Tariffing the services included in health basic package in an appropriate and scientific way using actual prices based on activity costing and local evidences.

Integrating of policies and precedents in a way that all insurance companies can access to a comprehensive electronic database of the insured information and their medical and health records and try to eliminate the overlap that is now existed in utilization of some packages by a single insured from different insurance organizations’ services.

The last subtheme in this axis, was Purchasing the benefit packages applying strategic purchasing through customized guidelines and creating the power of win–win contracting between purchasers and providers.

#### Health care provider requirements

This axis consists of four subthemes as follows: Determining the health and treatment necessities of the community according to their age, sex and epidemiological description of the diseases and presenting a cost-effective package in preventive, therapeutic, rehabilitation, promotive and palliative services.

Provision of the above services according to the population’s need and trying to achieve necessary credits for competing with other provider agencies in contracting the third party insurers.

Rules relating to implementation of health basic benefit package through creating the necessary measures, notifying mechanisms for universal and compulsory health and cooperating between all basic insurance organizations and providers was another subtheme in this axis.

The last subtheme was covered services emphasizing high risk groups, children and women and considering high priority services like infertility treatment and primary health care.

#### Citizen/population requirements

The last axis has two subthemes containing: first, expanded coverage of population that means increasing the coverage of population containing headed household, residents of villages/cities with less than 20,000 people, all uninsured Iranians and developing urban inpatient scheme to underserved poor populations and then, Insurance premium that focuses on collecting rational incremental insurance premiums as a per capita from all insured population along with considering the share of employees premiums that was paid by executive organizations as the agent of citizens.

### Discussion

Findings showed that three main requirements are mentioned in Iranian national documents with a growing trend in the last two decades consisting the third party insurer, health care provider and citizen/population requirements all of them need to be considered by policy makers to achieve UHC.

In the first axis, third party insurer, the structure of the insurance organization and the integration of policies must be considered as well as financing, tariffing and purchasing health basic benefit package. In this regard WHO recommended options, for low- and middle-income countries, for increasing the share of health from the general budget [26]. This recommendation is considered by Iranian policy makers so that the 2015 budget for the health sector increased by nearly 70% compared to 2014 [27]. However the sustainability and reallocation of these funds must be noticed in a way of achieving UHC. Typical instability of financial resources was found in Mongolia where more than 95% of the population was covered in 1996 after which, a decreasing trend of population coverage was experienced because of financial instability [28].

Emphasis on marked taxes is also one way to increase the share of health resources. This type of tax directly improves health outcomes through reducing demands for harmful goods [29]. This happened in South Korea [30] and Ghana [29] and almost 3.1% of the insurance fund was provided in South Korea in 2010. Moreover, health insurance policymakers in Nigeria [26] proposed tax bills to mobile phones as a way of financing health coverage. Such these issues must be considered as funding mechanisms helping third party insurers collect revenues and allocate them to the most needed health services to achieve UHC.

Another important topic in this axis is changing the structure of insurers. The experiences show that one basic measure for achieving UHC, especially in developing countries, is reduction of fragmentation of the insurance fund [29, 31, 32]. This concern can be disappeared through implementing a new structure and integration the insurers’ financial resources in an a single and uniform insurance organization as it is mentioned in Article 38 of the fifth 5-year development plan [33].

The second axis of the present results, health provider requirements, includes determining the necessities, provision and covered services and also rules relating to implementation health basic benefit package. In this regard, evidences show that by moving toward UHC, health systems have no way but to establish the basic health insurance package [13]. What is covered by insurance plans is important according to their ultimate effect on public health and financial protection [26]. An important issue is attending to all areas of services from promotion to palliative care [34]. So it is important for Iran to determine the services included in national basic benefit package in all levels according to the local needs. It is obvious that for obtaining this goal including inpatient and outpatient services could not be sufficient. This experience is confirmed by India, Kenya, and the Philippines which initially covered inpatient services, are now moving toward covering primary and preventive services, recognizing the fact that although outpatient services may be costly, they can lead to a greater impact on health outcomes [26].

Finally, the last axis, citizen/population requirements focused on expanding population and their insurance premiums. In this regard, evidences show different countries have adopted various policies achieving UHC; in South Korea and Vietnam, first the formal sector was covered and then, the informal sector and whole population was expanded [35]. On the other hand, India has allocated significant subsidies to the target population and has tried to expand financial protection through increasing tax revenues [36]. Furthermore, instead of considering multiple programs for different groups of population, some countries have implemented integrated coverage programs for all populations [37].

### Conclusion

Results show that Iranian policy makers should notice three axes of third party insurers, health providers and population of the country to prepare an appropriate basic benefit package based on local needs for all the people that can access with no financial barriers. In this way UHC can be assured in this developing country.

## Limitations

Documents alone are not able to express all the facts related to politics. It is recommended to apply multiple methods sequentially or simultaneously in another qualitative study. Obviously, application of documentations along with other data collection methods, as interviews or observations, can help researchers reach more comprehensive understanding.

## Data Availability

All data generated or analyzed during this study are included in this published article.

## Abbreviations

WHO: World Health Organization
UHC: universal health coverage
